# Improving the Therapeutic Potential of G-CSF through Compact Circular PEGylation Based on Orthogonal Conjugations

**DOI:** 10.1021/acs.biomac.3c00543

**Published:** 2023-08-28

**Authors:** Antonella Grigoletto, Valentina Marotti, Tommaso Tedeschini, Benedetta Campara, Ilaria Marigo, Vincenzo Ingangi, Gianfranco Pasut

**Affiliations:** † Department of Pharmaceutical and Pharmacological Sciences, University of Padova, Via Marzolo 5, 35131 Padova, Italy; ‡ Department of Surgery, Oncology and Gastroenterology, University of Padova, 35131 Padova, Italy; § Istituto Oncologico Veneto IOV − IRCCS, Via Gattamelata 64, 35128 Padova, Italy

## Abstract

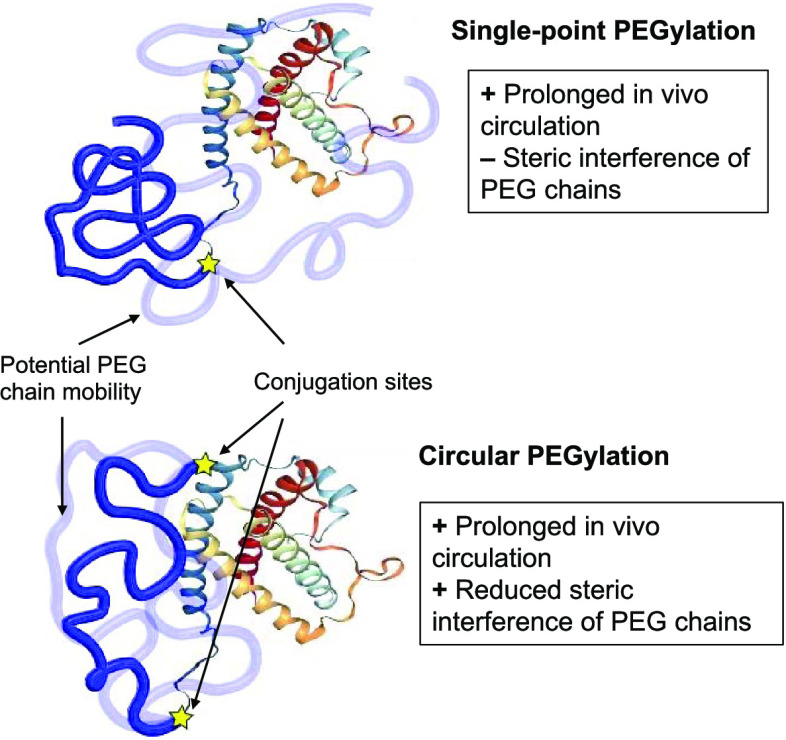

In this study, a circular conjugate of granulocyte colony-stimulating factor (G-CSF) was prepared by conjugating the two end-chains of poly(ethylene glycol) (PEG) to two different sites of the protein. For the orthogonal conjugation, a heterobifunctional PEG chain was designed and synthesized, bearing the dipeptide ZGln-Gly (ZQG) at one end-chain, for transglutaminase (TGase) enzymatic selective conjugation at Lys41 of G-CSF, and an aldehyde group at the opposite end-chain, for N-terminal selective reductive alkylation of the protein. The cPEG-Nter/K41-G-CSF circular conjugate was characterized by physicochemical methods and compared with native G-CSF and the corresponding linear monoconjugates of G-CSF, PEG-Nter-G-CSF, and PEG-K41-G-CSF. The results demonstrated that the circular conjugate had improved physicochemical and thermal stability, prolonged pharmacokinetic interaction, and retained the biological activity of G-CSF. The PEGylation strategy employed in this study has potential applications in the design of novel protein-based therapeutics.

## Introduction

In the past few decades, protein-based drugs produced through recombinant DNA techniques have gained exceptional roles in several therapies due to their advantageous combination of selective activity, potency, and safety. Recombinant human granulocyte colony-stimulating factor (rhG-CSF) is a hematopoietic cytokine approved in 1991 by the FDA (Filgrastim) for the treatment of chemotherapy-induced neutropenia.^1^ Human G-CSF is a glycoprotein mainly produced by the stromal cells of bone marrow during granulopoiesis. It mobilizes hematopoietic and progenitor stem cells and induces and regulates the proliferation, differentiation, and survival of granulocyte cells, especially neutrophils, which have a fundamental role in the initial immune response following bacterial and fungal infections.^2^ G-CSF interacts with its receptor, which is prevalently expressed on the surface of hematopoietic precursor cells and neutrophilic granulocytes.^3^ The rhG-CSF is used for the treatment of congenital and acquired neutropenia because it improves the granulocyte count in neutropenic patients^4^ and for the mobilization of hematopoietic stem cells in case of stem cell transplantation.^5^

The main limitation of G-CSF as a biological drug is its rapid kidney clearance due to its relatively low molecular weight (∼18.8 kDa). For this reason, it has been an ideal candidate for polymer conjugation, especially PEGylation, aiming to increase its hydrodynamic size for pharmacokinetic prolongation. Several chemical and enzymatic methods of conjugation have been proposed over the years,^6−13^ thus making G-CSF one of the most studied proteins for polymer conjugation. Currently, there are two PEGylated G-CSF conjugates in clinical practice. The first has been obtained by reductive alkylation at the protein N-terminus,^14^ while the second has been obtained by glycoPEGylation, a strategy that allows polymer coupling at the protein glycan moiety through an enzymatic method.^15,16^ Consequently, this protein is a perfect model for the evaluation and comparison of any new method of polymer conjugation.

**Figure 1:**
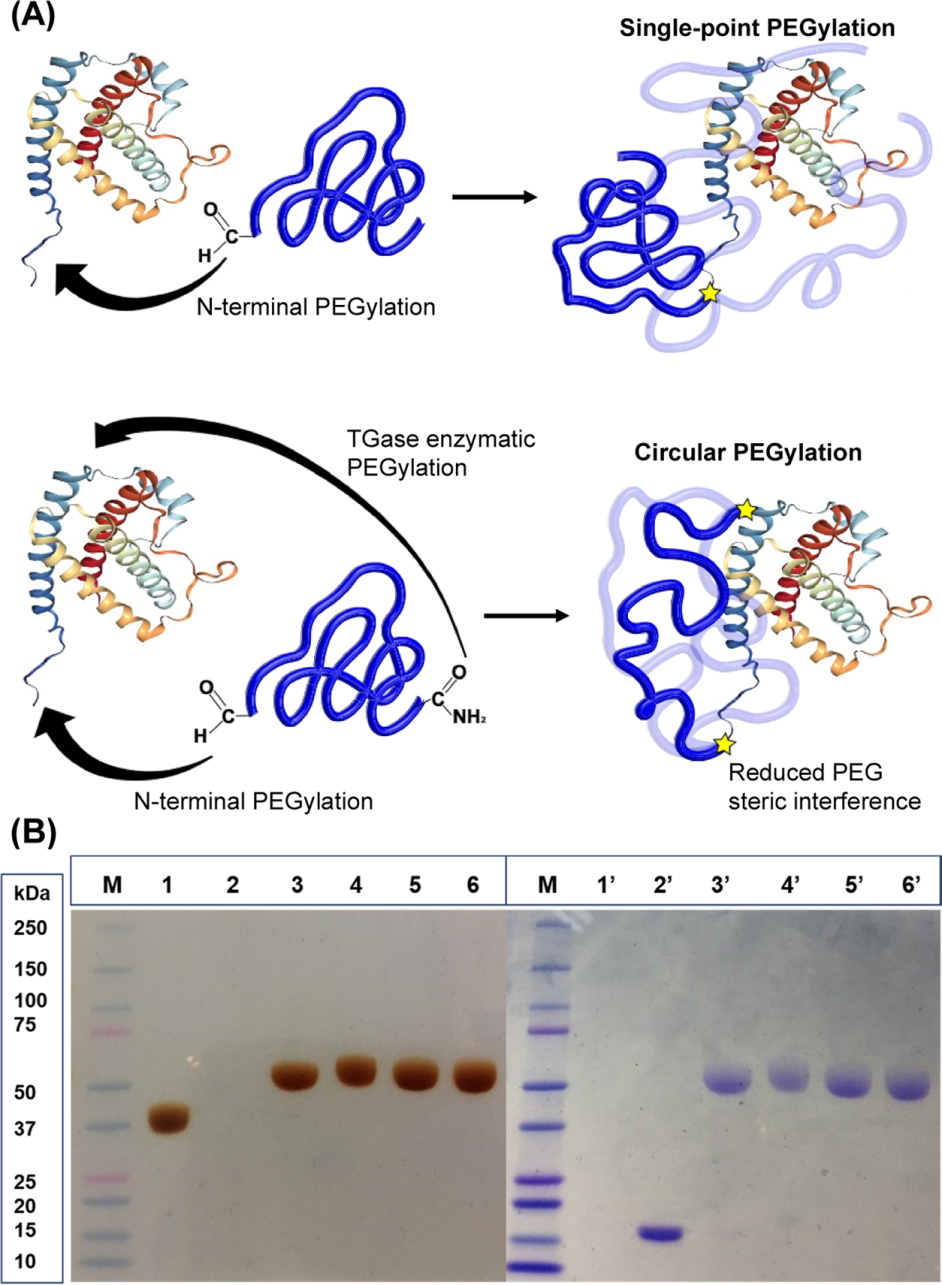
(A) Schematic representation of single-point PEGylation and circular PEGylation. (B) SDS-PAGE (4–15% gradient gel) attesting the purity of the conjugates. Lanes 1 and 1′: ZQG-PEG20kDa-aldehyde (≈37 kDa); Lanes 2 and 2′: G-CSF (≈18 kDa); Lanes 3 and 3′: ZQG-PEG-Nter-G-CSF (≈50 kDa); Lanes 4 and 4′: cPEG-Nter/K41-G-CSF (≈50 kDa); Lanes 5 and 5′: PEG-Nter-G-CSF (≈50 kDa); Lanes 6 and 6′: PEG-K41-G-CSF (≈50 kDa). The markers are in Lane M; Lanes 1–6 are referred to iodine staining for PEG detection and Lanes 1′–6′ are referred to Coomassie staining for protein detection, obtained from the same SDS-PAGE gel stained in sequence.

Here, we describe a site-selective approach of PEGylation based on the conjugation of both polymer end-chains to the same protein unit to achieve circular polymer conjugation (Figure 1A). The intent was to seek a more compact conformation of PEG over the protein surface, thus reducing its mobility and sprouting from the protein and, therefore, decreasing the polymer interference with the process of protein/receptor interaction. To reach this aim, two site-selective conjugation approaches have been performed to link the same PEG chain at the G-CSF N-terminus via reductive alkylation and at the G-CSF K41 via mTGase. The PEG required orthogonal reactivities for performing the two selective couplings without interferences, namely, an aldehyde on one side, for the reductive alkylation, and a carbobenzoxy-protected glutaminyl-glycine (ZQG) on the other side, for mTGase-mediated conjugation.

To the best of our knowledge, this is the first time that a circular PEGylation based on two site-selective conjugation approaches is applied and the results obtained warrant further investigations.

## Experimental Section

### Materials

The protein rhG-CSF was a kind gift of Sandoz (Ljubljana, Slovenia). PEG-aldehyde 20 kDa and PEG-NH_2_ 20 kDa were purchased from NOF Corporation (Tokyo, Japan). The H_2_N-PEG-COOH 20 kDa was purchased from JenKem Technology USA, Inc. (Plano, TX). Microbial TGase (mTGase), of *Streptomyces mobaraensis* origin (ACTIVA M), was provided by Ajinomoto Co. (Tokyo, Japan). Carbobenzoxy-l-glutaminyl-glycine and all of the chemicals and solvents were purchased from Merk (Darmstadt, Germany). Trypsin and Glu-C of sequencing grade were obtained by Thermo Fisher Scientific (Waltham, MA). Human G-CSF Instant ELISA Kit was purchased from Life Technologies (Waltham, MA). Mini-PROTEAN TGX precast gels 4–15% for SDS-PAGE were obtained from Bio-Rad (Milan, Italy).

### Analytical Methods

#### NMR Analysis

All ^1^H NMR spectra were recorded on a Bruker 400 MHz FT-NMR spectroscope equipped with a dual thermostated ^1^H/^13^C gradient probe and a Fourier 80 benchtop cryogen-f. TMS was used as internal standard.

#### UV–Vis Spectroscopy

Protein concentrations were determined spectrophotometrically on a Thermo Scientific Evolution 201 spectrophotometer (Waltham, MA). The concentration of proteins was evaluated by measuring the absorbance at 280 nm and using the following extinction coefficients: ε_G-CSF_ = 0.88 and ε_TGase_ = 1.89 mL cm^–1^ mg^–1^. The values of the absorbance at 280 nm were generated by ProtParam (http://www.expasy.org/tools/protparam.html).

The concentration of protein conjugates was determined by bicinchoninic acid colorimetric assay (BCA) as described elsewhere.^17^

#### Sodium Dodecyl Sulfate-Polyacrylamide Gel Electrophoresis (SDS-PAGE)

The conjugates were analyzed by SDS-PAGE following the Laemmli method.^18^ Electrophoresis was performed using gradient 4–15% Mini-PROTEAN TGX precast gels (Bio-Rad Laboratories Hercules, CA) loaded on a Mini-PROTEAN Tetra Cell obtained from Bio-Rad, and the runs were carried out using an Electrophoresis Power Plus300 power supply (Fisher Scientific, MI, IT) at 300 V and 60 mA. The gels were first stained with barium iodine for PEG detection^19^ and then with Coomassie Brilliant Blue R-250 for protein detection. Briefly, for the reversible iodine staining, the gels were soaked in 20 mL of perchloric acid (0.1 M), and after 15 min, the gel was transferred into 10 mL of a 5% (w/v) BaCl_2_ solution in 1 N HCl and 4 mL of a 1.27% (w/v) I_2_ and 2% (w/v) KI aqueous solution, yielding the appearance of PEG-containing bands in a few minutes. The staining solution was discarded, and the gels were incubated in water for 10–15 min. The iodine staining was then removed by the addition of 5 mg of ascorbic acid. After 10 min the gel was washed with water and, in turn, stained with a standard procedure for Coomassie blue staining of proteins.

#### Matrix-Assisted Laser Desorption/Ionization Time-of-Flight Mass Spectra (MALDI-TOF MS) Analysis

MALDI-MS data were obtained using a REFLEX time-of-flight instrument (4800 Plus MALDI-TO/TOF, AB Sciex, Framingham, MA) equipped with a SCOUT ion source, operating in the positive linear mode. Ions generated by a pulsed UV laser beam (nitrogen laser, λ 337 nm) are accelerated to 25 kV. The matrix, a saturated solution of sinapinic acid in water/ACN (50:50, v/v) with 0.1% TFA (v/v), was mixed with an equal volume of sample solution, and 1 μL of the resulting mixture was spotted on the MALDI target.

### Synthesis of ZQG-PEG-Acetal

**Scheme 1:**
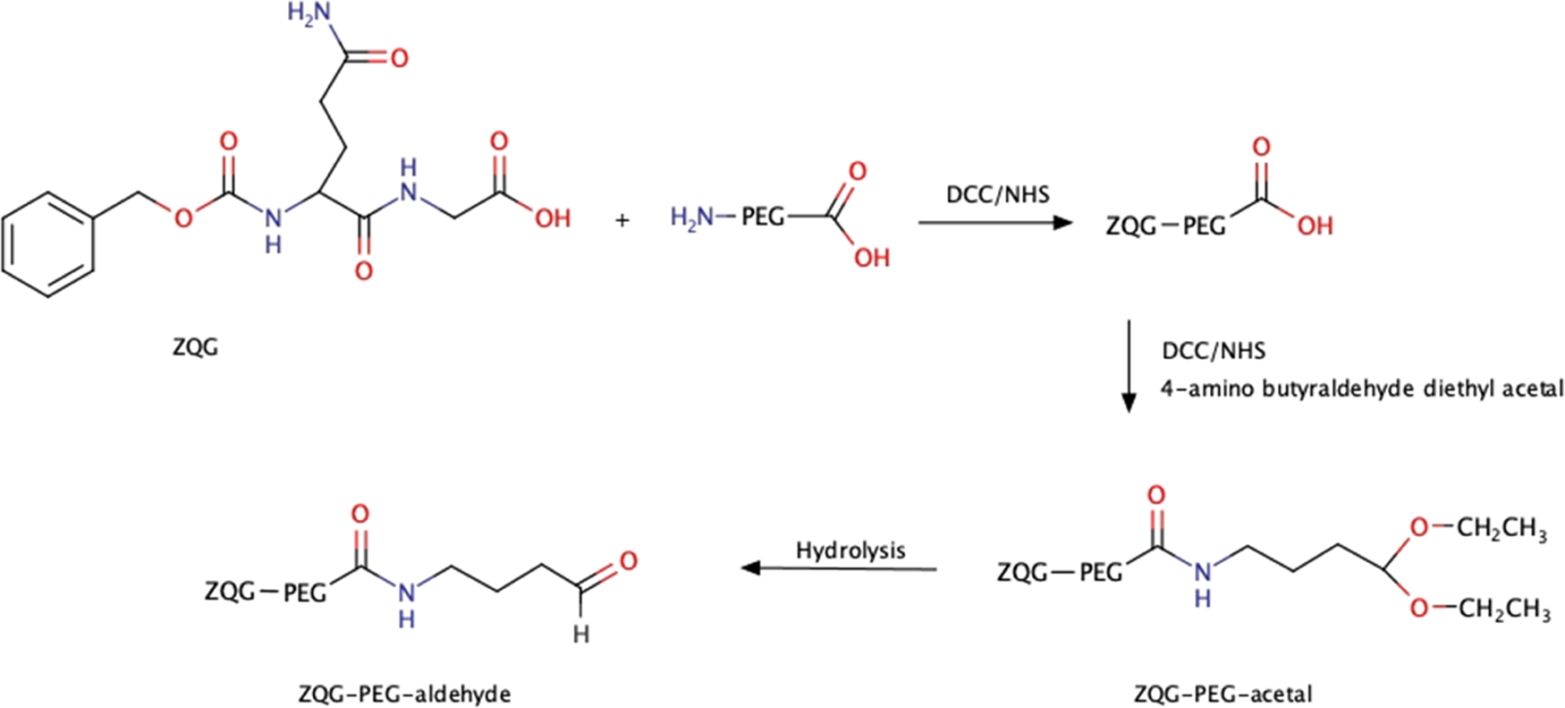
Synthesis of ZQG-PEG-Aldehyde

The preparation of ZQG-PEG-acetal was achieved in two steps, and the derivative was then hydrolyzed to ZQG-PEG-aldehyde just before G-CSF conjugation (Scheme 1).

The starting polymer NH_2_-PEG-COOH (MW 20 kDa) was first derivatized with ZQG at the amino group of the polymer. After purification and recovery, the product was modified with 4-amino butyraldehyde diethyl acetal at the carboxylic group on the other side chain of the polymer.

#### Synthesis of ZQG-PEG-COOH

515.6 mg (2.5 mmol) of *N*,*N*′-dicyclohexylcarbodiimide (DCC, MW 206.33 Da) and 143.8 mg (1.25 mmol) of *N*-hydroxysuccinimide (NHS, MW 115.09 Da) were added to 168.6 mg (0.5 mmol) of ZQG (MW 337.33 Da) previously dissolved in 5 mL of anhydrous DMSO. After 1 h, 500 mg (0.025 mmol) of NH_2_-PEG-COOH (MW 20 kDa) was added and 25 μL of Et_3_N was added. The reaction was left to stir at room temperature for 18 h, and the degree of derivatization was estimated by the TNBS-based test according to the Snyder and Sobocinski assays.^20^ Finally, 7 μL (0.075 mmol) of acetic anhydride (MW 102.09 Da, ρ 1.08 g/mL) was added to the mixture to quench the reaction. The absence of free amino groups was verified through the Snyder and Sobocinski assay. The solution was extensively dialyzed against a mixture of Milli-Q water and decreasing amounts of DMSO and for 18 h against Milli-Q water. The absence of ZQG was verified through RP-HPLC on an Agilent 1200 Series HPLC with online UV detection from Agilent Technologies (Santa Clara, CA). RP-HPLC analyses were performed using a Jupiter C18 column (250 × 4.6 mm^2^, 300 Å, 5 μm; Phenomenex, Torrance), eluted with H_2_O + 0.05% TFA (eluent A) and ACN + 0.05% TFA (eluent B) at a flow rate of 1.0 mL/min applying the following gradient of eluent B: 0′ 40%, 25′ 70%, 30′ 95%, 35′ 40% B. The absorbance was read at 226 nm. After lyophilization (yield: 432 mg, 86.4% w/w), the identity of the product ZQG-PEG_20 kDa_-COOH was evaluated by ^1^H NMR: (D_2_O, δ ppm) PEG: 4 (s, 2H), 3.7 (s, 1970H), ZQG: 7.41 (m, 5H), 5.13 (s, 2H).

#### Synthesis of ZQG-PEG-Acetal

ZQG-PEG_20 kDa_-COOH (400 mg, 0.02 mmol) was dissolved in 4 mL of anhydrous CH_2_Cl_2_, and 20.6 mg (0.1 mmol) of DCC (MW 206.33 Da) and 5.7 mg (0.05 mmol) of NHS (MW 115.09 Da) were added. After 1 h, 11.5 μL (0.06 mmol) of 4-amino butyraldehyde diethyl acetal (MW 161.24 Da, *d* 0.933 g/mL) was added and the pH of the solution was adjusted to 8. The reaction was stirred at room temperature for 18 h. The mixture was dropped into cold diethyl ether under stirring, and the obtained precipitate was washed with fresh diethyl ether to remove the excess of 4-amino butyraldehyde diethyl acetal. The polymer was dried under vacuum (yield: 342 mg, 85.5% w/w), and the absence of 4-amino butyraldehyde diethyl acetal was confirmed on thin-layer chromatography stained with 0.2% w/v ninhydrin in EtOH solution and using a solution of acetal as reference. The derivatization degree of the product ZQG-PEG_20 kDa_-acetal was determined by ^1^H NMR by comparing the integration values of the methylene hydrogens of acetal moiety with the signal of the monomers of PEG backbone: (D_2_O, δ ppm) PEG: 4 (s, 2H), 3.7 (s, 1970H), ZQG: 7.41 (m, 5H), 5.13 (s, 2H), 4-amino butyraldehyde diethyl acetal: 1.17 ppm (t, 6H). The molecular weight of ZQG-PEG_20 kDa_-acetal was determined by MALDI-TOF.

### Synthesis of cPEG-Nter/K41-G-CSF

The acetal moiety of ZQG-PEG_20 kDa_-acetal was hydrolyzed to the aldehyde group: 36 mg of ZQG-PEG_20 kDa_-acetal (1.8 μmol; degree of acetal modification 88.4 mol %) was dissolved in 360 μL of 25 mM H_3_PO_4_ pH 2.1 and stirred for 2 h at 60 °C. After cooling to room temperature, ZQG-PEG_20 kDa_-aldehyde was incubated with G-CSF at a final protein concentration of 2 mg/mL as follows: to a stock solution of G-CSF (∼4.6 mg/mL, 0.32 μmol) in 10 mM sodium acetate, 5% (w/v) sorbitol pH 4.6, ZQG-PEG_20 kDa_-aldehyde was added after diluting its solution with the same buffer (G-CSF/ZQG-PEG_20 kDa_-aldehyde ratio 1:5). After 1 h under stirring at 25 °C, 100 equiv of NaCNBH_3_ (with respect to the protein) were added. The mixture was incubated at 25 °C and 300 rpm and, after 24 h, was analyzed by RP-HPLC on an Agilent 1200 Series HPLC with online UV detection from Agilent Technologies (Santa Clara, CA). RP-HPLC analyses were performed using a Jupiter C18 column (250 × 4.6 mm^2^, 300 Å, 5 μm; Phenomenex, Torrance), eluted with H_2_O containing 0.05% TFA (eluent A) and ACN containing 0.05% TFA (eluent B) at a flow rate of 1.0 mL/min applying the following gradient of eluent B: 0′ 40%, 25′ 70%, 30′ 95%, 35′ 40% B. The absorbance was recorded at 226 nm. Gly–Gly (3 equiv with respect to the polymer) was added to stop the reaction. After 1 h, the conjugate ZQG-PEG-Nter-G-CSF was purified by cation exchange chromatography (CEX) using a TSKgel SP-5PW column (7.5 × 75 mm^2^; 10 μm) operating at a flow rate of 1.0 mL/min and registering the absorbance at 280 nm (buffer A: 10 mM sodium phosphate pH 4.7 and buffer B: 100 mM sodium phosphate, 100 mM sodium chloride pH 4.85; gradient B%: 0′ 5%, 5′ 5%, 65′ 100%, 80′ 100%, 85′ 5%). The separation was performed on a Shimadzu HPLC with CBM-20Alite system controller, LC-20AT pump, and SPD-10A VP detector. The peak of the conjugate was collected, concentrated, and buffer-exchanged to 10 mM sodium acetate, 5% (w/v) sorbitol pH 4.6 with Amicon Ultra Centrifugal filters (cutoff 10 kDa; Millipore, Merck). ZQG-PEG-Nter-G-CSF quantification was performed through the BCA colorimetric assay as reported above, and the purity of the conjugate was verified through SDS-PAGE.

To complete the synthesis, mTGase was used to tether the ZQG end of the PEG conjugated to G-CSF: ZQG-PEG-Nter-G-CSF (0.16 μmol) was buffer-exchanged to 10 mM sodium phosphate pH 7.2 with Amicon Ultra Centrifugal filters and mTGase was added at an enzyme/substrate (E/S) ratio of 1:25 (w/w), previously solubilized in 0.1 M sodium phosphate buffer pH 7.2 and quantify by UV spectroscopy. The G-CSF concentration was 1 mg/mL. After 18 h at 25 °C under stirring, the reaction was stopped by the addition of a solution of *N*-ethylmaleimide (NEM) at a molar ratio of 1.25 equiv with respect to mTGase. The mixture was first purified by size exclusion chromatography (SEC) on an AKTA FPLC (Amersham Biosciences) using a Superdex 200 Increase 10/300 GL column (30 cm × 10 mm, 8.6 μm particle size, GE Healthcare) eluting with PBS pH 7.4 at 0.5 mL/min and measuring the absorbance at 280 nm. The peak corresponding to a mixture of the conjugates ZQG-PEG-Nter-G-CSF and cPEG-Nter/K41-G-CSF was collected, concentrated, and buffer-exchanged to 10 mM sodium acetate pH 4.7. The conjugates were separated through cation exchange chromatography using the conditions reported above. Finally, cPEG-Nter/K41-G-CSF was concentrated, buffer-exchanged to 10 mM sodium acetate, 5% (w/v) sorbitol pH 4.6 with Amicon Ultra Centrifugal filters (cutoff 10 kDa), and quantified by BCA. The conjugate was characterized through SDS-PAGE, MALDI-TOF, and SEC.

### Trypsin In-Gel Digestion of cPEG-Nter/K41-G-CSF and LC–MS^E^ Analyses

Native protein, the intermediate ZQG-PEG-Nter-G-CSF, and cPEG-Nter/K41-G-CSF (30 μg) were analyzed by SDS-PAGE. The respective slices were isolated and washed three times with 200 μL of a 0.1 M NH_4_HCO_3_/ACN 50:50 (v/v) mixture at pH 7.8 for 10 min. After dehydration with ACN for 15 min, gel slices were completely dried in a speed-vac system (Thermo Fisher Scientific, Waltham, MA). Native and conjugated proteins were reduced by incubation with 200 μL of 5 mM tris(2-carboxyethyl)phosphine (TCEP) in 0.1 M NH_4_HCO_3_ pH 7.8 for 10 min at 60 °C and alkylated by adding 200 μL of 55 mM iodoacetamide (IAA) in 0.1 M NH_4_HCO_3_ pH 7.8 for 15 min at 37 °C in the dark. Gel slices were washed twice with a 0.1 M NH_4_HCO_3_/ACN 50:50 (v/v) mixture at pH 7.8 for 10 min, dehydrated with ACN for 15 min and again completely dried. 50 μL of trypsin protease (Thermo Fisher Scientific, Waltham, MA; 10 μg/mL in 0.1 M NH_4_HCO_3_ pH 7.8) were added and the digestion reaction was let to proceed overnight at 37 °C and 300 rpm in a thermomixer (Eppendorf, Hamburg, Germany). Formic acid to a final concentration of 2.5% (v/v) was added, and the obtained peptide solutions were desalted by PepClean C18 Spin columns (Thermo Fisher Scientific, Waltham, MA) following the manufacturer’s instructions. The digested mixtures were analyzed using a UPLC-QTOF system. The ACQUITY UPLC H-Class (Waters, Milford, MA) was equipped with an AdvanceBio Peptide Map Guard (2.1 × 5 mm^2^, 2.7 μm; Agilent) and AdvanceBio Peptide Mapping column (150 × 2.1 mm^2^, 2.7 μm; Agilent), maintained at 32 °C, flow rate 0.2 mL/min, detection at 280 nm, eluted with a solvent gradient of water/ACN both containing 0.1% formic acid. Gradient 2′ – 2% ACN, 38′ – 65% ACN, 40′ – 98% ACN, 43′ – 98% ACN, 44′ – 2%. The Xevo G2-S QTof (Waters) was operated in the ESI positive ion, resolution mode, with a detection window between 50 and 2000 m/z. Analyses were performed at a capillary voltage of 1.5 kV, at a cone voltage of 30.0 V, and source offset of 80 V. MS^E^ acquisition was performed by alternating two MS data functions: one for the acquisition of the peptide mass spectra with the collision cell at low energy (6 eV) and the other for the collection of the peptide fragmentation spectra with the collision cell at elevated energy (linear ramp 20–40 eV). Analyses were performed with LockSpray using a solution of 1 ng/μL LeuEnk MS in 50:50 (v/v) water/ACN containing 0.1% formic acid, sampled every 45 s. MS^E^ data were processed with MassLynx and BiopharmaLynx 1.3.4 Software (Waters) setting trypsin as the digest reagent and 2 missed cleavages. The MS ion intensity threshold was set at 250 counts, and the MS^E^ threshold was set to 100 counts. Both the MS masses-match tolerance and the MS^E^ mass-match tolerance were set to 15 ppm.

### Trypsin Digestion of cPEG-Nter/K41-G-CSF and MALDI-TOF Analyses

cPEG-Nter/K41-G-CSF was incubated with trypsin in ammonium bicarbonate pH 8.0, at 37 °C and 300 rpm in a thermomixer. Trypsin was added at an enzyme/substrate (E/S) ratio of 1:100 (w/w), and the final concentrations of G-CSF and trypsin were 0.2 mg/mL and 2 μg/mL, respectively. After 18 h, the reaction mixture was analyzed by MALDI-TOF.

### N-Terminal PEGylation of G-CSF with PEG_20 kDa_-Aldehyde

PEG-Nter-G-CSF was prepared as reported elsewhere^8^ to be used for a direct comparison against the cPEG-Nter/K41-G-CSF. Briefly, 3 equiv of monomethoxy PEG-aldehyde 20 kDa previously solubilized in 10 mM sodium acetate, 5% (w/v) sorbitol pH 4.6 were added to a stock solution of G-CSF (∼4.6 mg/mL, 0.21 μmol) in the same buffer. After stirring for 1 h at 25 °C, NaCNBH_3_ was added (100 equiv with respect to the protein); the final G-CSF concentration was 3 mg/mL. The mixture was incubated at 25 °C under stirring for 24 h, and it was monitored by RP-HPLC with a Jupiter C18 column (250 × 4.6 mm^2^, 300 Å, 5 μm; Phenomenex, Torrance, CA), eluted with H_2_O + 0.05% TFA (eluent A) and ACN + 0.05% TFA (eluent B) at a flow rate of 1.0 mL/min applying the following gradient: from 40 to 70% of ACN in 25 min. The absorbance was read at 226 nm. Gly–Gly (3 equiv with respect to the polymer) was added to stop the reaction. After 1 h, PEG-Nter-G-CSF was purified by cation exchange chromatography using a TSKgel SP-5PW column (7.5 × 75 mm^2^; 10 μm) operating at a flow rate of 1.0 mL/min and registering the absorbance at 280 nm (buffer A: 10 mM sodium phosphate pH 4.7 and buffer B: 100 mM sodium phosphate, 100 mM sodium chloride pH 4.85; gradient B%: 0′ 5%, 5′ 5%, 65′ 100%, 80′ 100%, 85′ 5%). The conjugate was concentrated and buffer-exchanged to 10 mM sodium acetate, 5% (w/v) sorbitol pH 4.6 with Amicon Ultra Centrifugal filters (cutoff 10 kDa; Millipore, Merck). G-CSF concentration was determined by UV spectroscopy and BCA.

### mTGase-Mediated PEGylation of G-CSF with PEG_20 kDa_-ZQG

PEG-ZQG was prepared as reported elsewhere.^21^ 1-Ethyl-3-(3-(dimethylamino)propyl)carbodiimide (EDC, MW 191.7 Da) (38.3 mg, 200 μmol) and 1-hydroxy benzotriazole (HOBT, MW 135.13 Da) (13.5 mg, 100 μmol) were added to 16.8 mg (50 μmol) of ZQG (MW 337.33 Da) previously dissolved in 2.5 mL of a 0.1 M borate buffer/ACN (3:2) mixture at pH 8.0. After 1 h, 250 mg (12.5 μmol) of PEG-NH_2_ (MW 20 kDa) was added, and the pH of the solution was adjusted to 8. The reaction was let to proceed for 18 h at room temperature under stirring, and the degree of derivatization was estimated by the Snyder and Sobocinski assays. Finally, 2.52 mg (25 μmol) of succinic anhydride (MW 100.08 Da) was added to the mixture to quench the reaction and the absence of free amino groups was verified using the TNBS assay. ACN was evaporated under vacuum and the remained solution was dialyzed for 24 h against Milli-Q water. After lyophilization, PEG_20 kDa_-ZQG identity was evaluated by ^1^H NMR: (D_2_O, δ ppm) PEG: 4 (s, 2H), 3.7 (s, 1970H), ZQG: 7.41 (m, 5H), 5.13 (s, 2H).

PEG-K41-G-CSF was prepared as reported elsewhere^11^ and used for the comparison with cPEG-Nter/K41-G-CSF. Briefly, G-CSF (∼4.6 mg/mL, 0.21 μmol) in 10 mM sodium acetate buffer pH 4.7, 5% (w/v) sorbitol was diluted with 10 mM sodium phosphate pH 7.2, and 20 equiv of PEG-ZQG (5.3 μmol; degree of ZQG derivatization 80 mol %) were added, previously dissolved in 10 mM sodium phosphate pH 7.2, in order to reach a final G-CSF concentration of 2 mg/mL. mTGase, solubilized in 0.1 M sodium phosphate buffer pH 7.2, was added at an E/S ratio of 1:50 (w/w) and the reaction was left to react under stirring for 18 h at 25 °C. After stopping the reaction with NEM (1.25 equiv with respect to mTGase), the buffer was exchanged to 10 mM sodium acetate pH 4.7 and PEG-K41-G-CSF was purified as reported for PEG-Nter-G-CSF.

### Circular Dichroism (CD) Analysis

Far-UV circular dichroism spectra were measured on a Jasco J-810 spectropolarimeter equipped with a Peltier temperature control unit. G-CSF and its derivatives were analyzed in 10 mM acetate, 5% sorbitol pH 4.6 at a protein concentration of 0.1 mg/mL. The spectra were collected over the wavelength range of 200–250 nm at 25 °C with an average of 3 scans, and the data at each wavelength were averaged for 8 s. The sample cell path length was 1 mm. The CD data were converted to mean residue ellipticity, expressed in deg cm^2^ dmol^–1^ by applying the formula Θ = Θ_obs_(MRW)/10*L*[*C*], where Θ is the observed ellipticity in degrees, MRW is the mean residue weight of the protein, [*C*] is protein concentration in mg/mL, and *L* is the optical path length in centimeters.

### Thermal Denaturation Studies

The thermal denaturation was carried out on the same samples used for CD analysis recording the decrease of the ellipticity signal at 222 nm as a function of the temperature. Thermal unfolding experiments were performed on a 0.1 cm cell path length by heating the samples from 25 to 90 °C at a rate of 2 °C/min. CD spectra in the range of 200–250 nm were then collected at 25 °C after heating to 90 °C.

### Pharmacokinetic Study in Rats

Pharmacokinetic profiles of G-CSF and G-CSF conjugates (cPEG_20k_-Nter/K41-G-CSF, PEG_20k_-Nter-G-CSF, and PEG_20k_-K41-G-CSF) were determined in female Sprague–Dawley rats weighing between 150 g and 160 g (3 animals per group). Samples were prepared in PBS pH 7.4 and were intravenously injected at the dose of 100 μg/kg (G-CSF equivalent) in the lateral tail vein. Anesthesia was performed with 5% isoflurane gas mixed with O_2_ in enclosed cages. Blood samples were collected on anesthetized rats by incision from the tail at predetermined time points and centrifuged at 1500*g* for 20 min. G-CSF equivalent concentrations in serum were quantified using a Human G-CSF Instant ELISA Kit (Life Technologies) by using the corresponding conjugates as standard. Pharmacokinetic data were elaborated using 2.0 PkSolver software by applying a bi-compartmental model. For each sample, a dedicated calibration curve was built using the testing conjugate.

### Pharmacodynamics Study of PEG-G-CSF Conjugates

The effect of conjugates on immune cell counts was evaluated in vivo in C57BL/6 female mice of 8 weeks (18–20 g) purchased from Charles River Laboratories.

cPEG-Nter/K41-G-CSF, PEG-Nter-G-CSF, and PEG-K41-G-CSF were injected subcutaneously as a single dose of 1 mg/kg of G-CSF equivalent, while native G-CSF was administrated daily at the dose of 0.14 mg/kg (4 mice per group). Vehicle solution (PBS) was used as control (CTRL). Myeloid cell (granulocytes and monocytes) accumulation was followed for 7 days at predetermined time points. Blood samples were collected at days 1, 3, and 5, and at day 7, after blood collection, the mice were sacrificed, and their spleens were harvested to be analyzed. The quantification of myeloid cell sub-populations in the blood or the spleen was performed by FACS analysis after staining with specific markers and defined on live cell gate as myeloid cells (CD11b+); granulocytes (CD11b+Ly6CintLy6G+); monocytes (CD11b+Ly6ChighLy6G−). Cytofluorimetric data were acquired with a BD LSR II flow cytometer and analyzed by FlowJo software. Injected samples were prepared using sterile water and resulted to be negative to the LAL test (endotoxin levels < 5 EU/mL).

### Ethics Statement

The study protocol was approved by the Ethics Committee of the University of Padova and the Italian Ministry of Health. The animals were handled in compliance with Italian Legislative Decree 116/92 guidelines and the “Guide for the Care and Use of Laboratory Animals” by the National Research Council of the National Academies.

## Result and Discussion

### Synthesis of ZQG-PEG-Acetal

ZQG-PEG_20 kDa_-acetal was synthesized starting from a heterobifunctional NH_2_-PEG-COOH through a two-step procedure, first involving the modification of the amino group of the starting PEG with the dipeptide ZQG, following the derivatization of the carboxylic end with 4-amino butyraldehyde diethyl acetal. The degree of derivatization with ZQG was 91 mol %. The absence of free ZQG was assured by monitoring the dialysis through RP-HPLC. The identity of the ZQG-PEG-COOH derivative was confirmed by ^1^H NMR (Figure S1B) and compared to the unmodified NH_2_-PEG-COOH (Figure S1A). In the second step, ZQG-PEG-COOH was reacted with 4-amino butyraldehyde diethyl acetal. The absence of 4-amino butyraldehyde diethyl acetal was verified by the ninhydrin test on TLC and the exact degree of derivatization was calculated from ^1^H NMR spectroscopy (Figure S1C). Acetal modification was calculated to be 88.4 mol %. The final PEG was characterized through MALDI-TOF and resulted in a molecular weight of about 21.1 kDa (Figure S2).

### Circular Site-Specific PEGylation of G-CSF

The circular conjugate cPEG-Nter/K41-G-CSF was synthesized by exploiting two different site-selective PEGylation strategies to obtain a homogeneous product. In the first step, G-CSF was selectively modified at the N-terminal residue by reductive alkylation, exploiting the aldehyde functionality of the polymer ZQG-PEG-aldehyde. The selectivity of this strategy relies on the difference between the α-amino group’s p*K*_a_ (6–7) of the N-terminal residue and the ε-amino group’s p*K*_a_ (9–10) of the lysine residues. At pH 4.6, the ε-amino groups are not reactive, differently, the α-amino group owing to its lower p*K*_a_ is still reactive, thus allowing the modification of G-CSF at the N-terminal residue. In the second step, mTGase-mediated PEGylation was performed between the other end of conjugated PEG and the lone lysine residue of G-CSF, which is a substrate of mTGase. This enzyme is a widely employed tool for obtaining site-selective monoconjugates in which the polymer is conjugated at specific glutamine or lysine residues based on the PEG used.^6,11,22,23^ In this case, the use of a PEG bearing the ZQG dipeptide, which is a Gln-containing mTGase substrate, has led to the derivatization of G-CSF at a Lys residue. As previously demonstrated,^1,24^ mTGase has specific substrate requirements for lysines, which means these amino acids must be inserted into flexible regions of the protein sequence. Only under such conditions can they reach the catalytic sites of the enzyme, following a similar pattern to that observed for glutamines.^24,25^ G-CSF presents four lysines, with the first three (Lys17, Lys24, and Lys35) located in the first α helix of the protein, resulting in a rigid conformation. In contrast, the fourth lysine (Lys41) is inserted in the loop between the first and second α helix, characterized by an increased B-factor value indicating peptide flexibility.^11^ Furthermore, Lys35 is preceded by Glu34, whose negative charge prevents interaction with the catalytic sites of TGase.^11^

The selective nature of TGase toward specific lysines has also been demonstrated with other proteins that contain multiple lysine residues. For instance, interferon α-2a possesses 10 lysines, among which Lys164 is predominantly recognized by TGase, with minor modifications (4.7%) occurring at Lys31 as a second site of conjugation forming the biconjugate.^26^

In the presented conjugation scheme, we initiate the process by coupling ZQG-PEG_20 kDa_-aldehyde to the N-terminus of the protein, followed by the enzymatic reaction to close the PEG circle. This chosen protocol offers several advantages. For instance, in TGase-mediated reactions, a substantial molar excess of PEG reagents (usually in the range of 10–50 fold relative to the protein) is necessary to avoid the formation of protein concatemers resulting from interprotein reactions between lysines and glutamines. Specifically, in the case of G-CSF, Lys41 and Gln135 are substrates of TGase. Without a proper excess of a PEG reagent, the formation of G-CSF concatemers becomes a concern. However, coupling the PEG to the N-terminus of G-CSF first overcomes this issue. The subsequent TGase-mediated reaction benefits from having the ZQG substrate, located at the opposite end of the PEG chain, in close proximity to Lys41 of the protein, thereby ensuring a high virtual concentration of the ZQG substrate near Lys41. Furthermore, the steric hindrance imposed by a 20 kDa PEG linked to the protein prevents the formation of the undesired PEG-G-CSF-Lys41-Q135-G-CSF-PEG dimer, leading to improved reaction yields.

Lastly, it is worth noting that if ZQG-PEG_20 kDa_-acetal were conjugated first, the acetal group would be deprotected by the acidic conditions during the purification step, posing the potential risk of undesired side reactions.

In the current study, the so-formed cPEG-Nter/K41-G-CSF was prepared with the focus to examine the outcomes of circular PEGylation on physicochemical characteristics, thermal stability, pharmacokinetic profile, and activity of the conjugate. cPEG-Nter/K41-G-CSF was also compared with the native protein and with the corresponding linear monoconjugates of G-CSF obtained by N-terminal and Lys41 PEGylation, by using mPEG-aldehyde 20 kDa and mPEG-ZQG 20 kDa, respectively.

The conjugation reaction of G-CSF with ZQG-PEG_20 kDa_-aldehyde was analyzed by RP-HPLC (Figure S3), resulting in a conversion yield of 73% based on the comparison of peak areas of G-CSF and the conjugate. At pH 4.6, the aldehyde of the polymer reacts with the N-terminal α-amino group of the protein, yielding the intermediate monoconjugate ZQG-PEG-Nter-G-CSF. The reaction was purified from the unreacted protein and the excess of ZQG-PEG-aldehyde by cation exchange chromatography (Figure S4). Before proceeding to the second step of the synthesis, the purity of ZQG-PEG-Nter-G-CSF was verified by SDS-PAGE. As shown in Figure 1B (lanes 3 and 3′), the formation of the conjugate was confirmed, and the product resulted to be purified from unreacted protein, di-PEGylated species, and free PEG. The intermediate conjugate had an apparent molecular weight of 50 kDa owing to the coordination of water molecules by the PEG chain. The absence of free PEG was established by iodine staining (Figure 1B).

**Figure 2:**
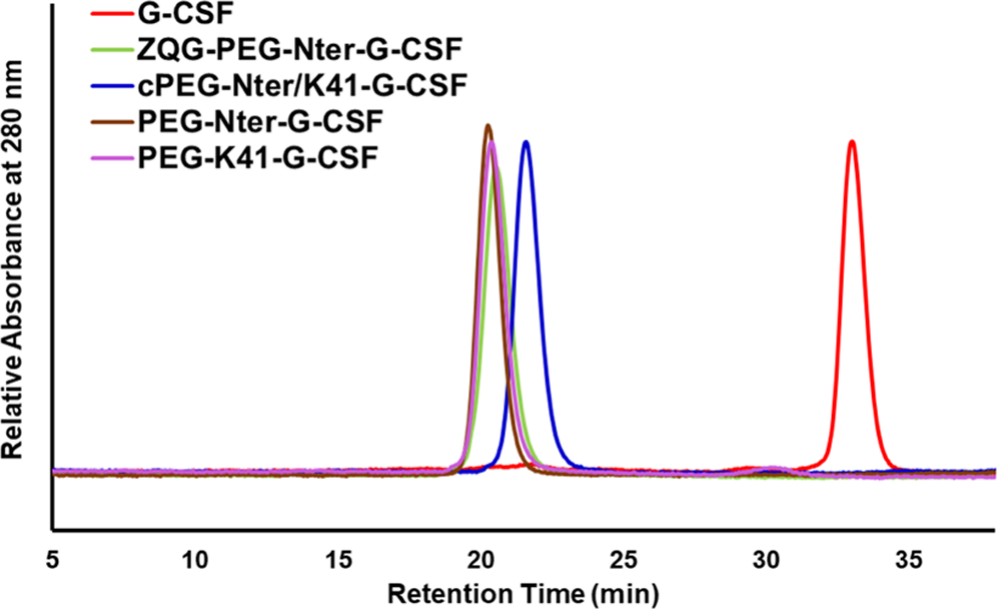
SEC-FPLC profiles of G-CSF, ZQG-PEG-Nter-G-CSF, cPEG-Nter/K41-G-CSF, PEG-Nter-G-CSF and PEG-K41-G-CSF.

The enzyme mTGase was used to close the PEG ring on G-CSF. mTGase formed, as side products, crosslinked conjugates owing to the concatenation of 2–3 units of ZQG-PEG-Nter-G-CSF, which were separated from the monoconjugates by SEC. The monoconjugates peak corresponded to a mixture of the mono-derivative ZQG-PEG-Nter-G-CSF (unreacted starting conjugate) and the desired circular conjugate. cPEG-Nter/K41-G-CSF was finally separated from ZQG-PEG-Nter-G-CSF through CEX (Figure S5). The purity of the circular conjugate was characterized by SDS-PAGE (Figure 1B, lanes 4 and 4′) and SEC-FPLC (Figure 2). cPEG-Nter/K41-G-CSF resulted purified from unreacted protein, from monoconjugate ZQG-PEG-Nter-G-CSF and free PEG. The results from SEC showed that ZQG-PEG-Nter-G-CSF, PEG-Nter-G-CSF, and PEG-K41-G-CSF presented a similar hydrodynamic volume (*T*_R_ 20.4 min), while cPEG-Nter/K41-G-CSF exhibited a reduced hydrodynamic volume (*T*_R_ 21.6 min). This behavior could be probably attributed to the final shape of the circular conjugate that, in solution, resulted more compact with respect to the linear conjugates.

The MALDI-TOF spectrum of cPEG-Nter/K41-G-CSF (Figure S6) has revealed a molecular weight (MW) of 39.9 kDa, corresponding to the sum of the MWs of the protein (18.8 kDa) and the polymer (21.1 kDa).

### Identification of the PEGylation Sites of Circular PEG-G-CSF

**Figure 3:**
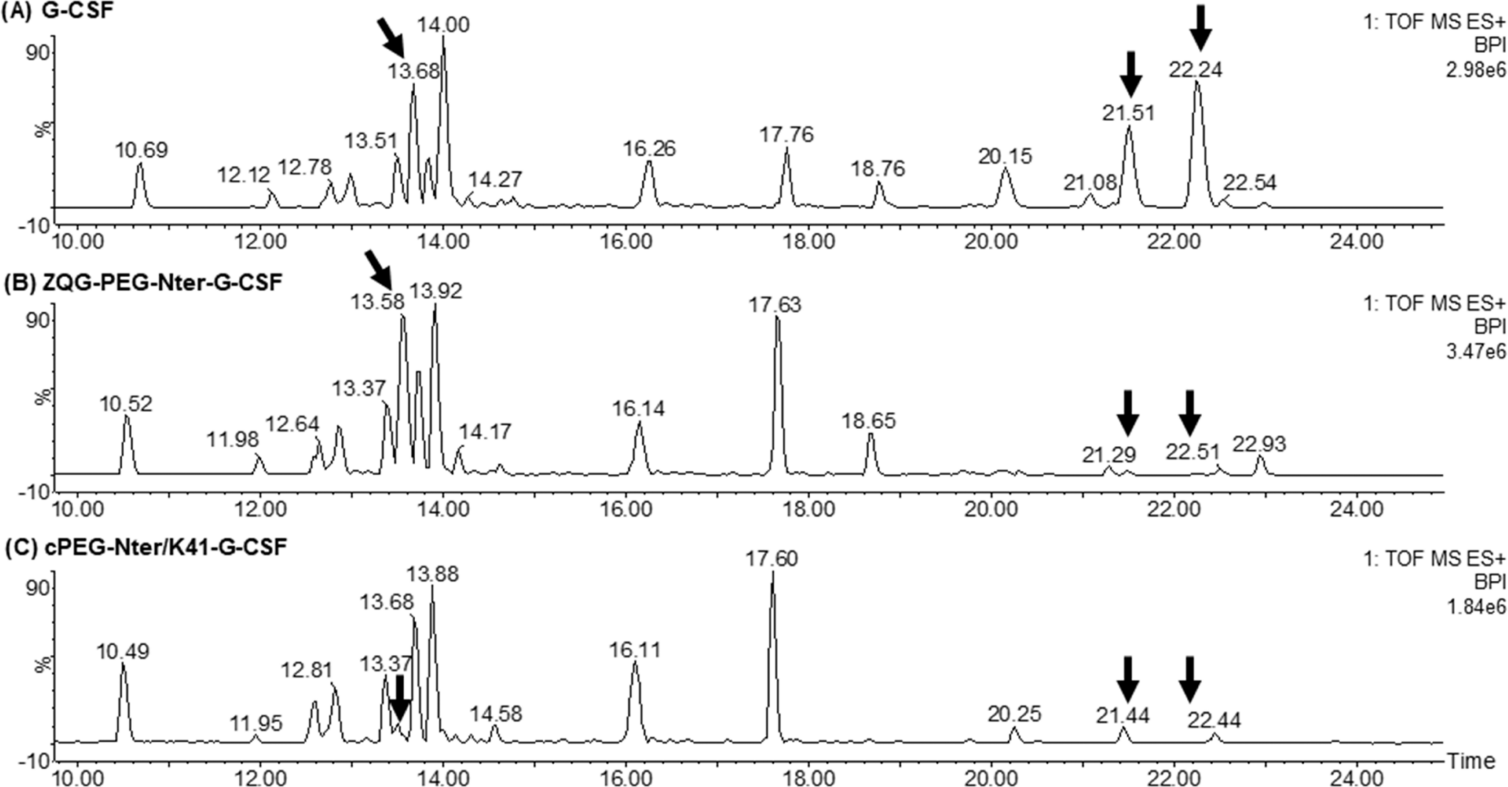
BPI chromatograms, extracted from MassLynx, generated in the LC–MS^E^ analyses of G-CSF (A), ZQG-PEG-Nter-G-CSF (B), and cPEG-Nter/K41-G-CSF (C) digested with trypsin protease. Almost every peak was matched to a peptide fragment. Peaks corresponding to peptide 1–17 (around 22.2 min), peptide 1–17 (ox) (around 21.5 min), and peptide 36–41 (around 13.6 min) are indicated by the arrows.

To identify the G-CSF residues that were specifically modified with PEG by circular PEGylation, cPEG-Nter/K41-G-CSF, ZQG-PEG-Nter-G-CSF, and G-CSF, as reference, were digested with trypsin protease under the same conditions. The peptide mixtures were analyzed by UPLC-MS (Figure 3), and the LC–MS^E^ raw data were processed using BiopharmaLynx (Waters). LC–MS^E^ peptide mapping of all of the digested samples resulted in a 39.4% of sequence coverage and the identity of the found peptides was confirmed by b/y fragment ions (Tables S1–S3). The obtained sequence coverage depends on trypsin cuttings. In fact, the digestion of G-CSF with trypsin forms the 42–147 fragment of 11.3 kDa that was undetected by the ESI-LC-MS^E^ method used. However, this fragment was not relevant to the analysis. In accordance with previous studies, the significative peptides for the identification of PEG modification in the circular conjugate are peptides 1–17 and 36–41. As shown in the LC–MS^E^ chromatogram of the digested native G-CSF (Figure 3A) and as verified by MS spectra (Figure S7), the peptide 1–17 (1785.97 Da) was eluted at 22.24 min, the peptide 1–17 with the oxidation at the methionine residue (1801.97 Da) was eluted at 21.51 min and was coeluted with peptide 149–167, and peptide 36–41 (754.37 Da) was eluted at 13.68 min. The masses and retention times of all of the detected fragments of native G-CSF are reported in Table S1. The LC–MS^E^ chromatogram of the proteolysis mixture relative to ZQG-PEG-Nter-G-CSF conjugate (Figure 3B) displayed the presence of the peak of peptide 36–41 at 13.58 min, while at 22.16 and 21.47 min, the peptides 1–17 and 1–17 (ox) have been identified with a very low intensity of the *m*/*z* signals, corresponding to 1.06 and 0.74% of the total intensity of the peptides, respectively. In the LC–MS^E^ chromatogram of the digested cPEG-Nter/K41-G-CSF (Figure 3C), the peaks relative to peptides 1–17 (22.15 and 21.44 min for the oxidized form) and 36–41 (13.54 min) were found with a very low intensity of 0.86, 0.67, and 0.1%, respectively, comparing to the total intensity of the peptides. The mass values of the peptides recorded for the PEGylated G-CSF were very similar to the theoretical expected ones (Tables S2 and S3). In conclusion, the mass fingerprinting analysis (Figure 3) demonstrated the disappearance of peptides 1–17 for ZQG-PEG-Nter-G-CSF and of peptides 1–17 and 36–41 for the circular conjugate, following conjugation with ZQG-PEG-aldehyde, confirming the circular PEGylation of G-CSF at the level of N-terminal and Lys41 residues.

As the PEGylated peptides cannot be detected with the ESI-LC-MS^E^ method used, the identification of the digested product (F1)-PEG-(F5-6) formed by the peptide F1 (1–17), the polymer, and the peptides F5 (36–41) and F6 (42–147) was evaluated by MALDI-TOF following the incubation of cPEG-Nter/K41-G-CSF with trypsin. The MALDI-TOF spectrum (Figure S8) has revealed a molecular weight of 34.9 kDa, corresponding to the sum of the MWs of peptide F1 (1–17, 1785.97 Da), polymer (21.1 kDa), peptide F5 (36–41, 754.37 Da), and peptide F6 (42–147, 11.29 kDa).

### Circular Dichroism (CD) Analysis

**Figure 4:**
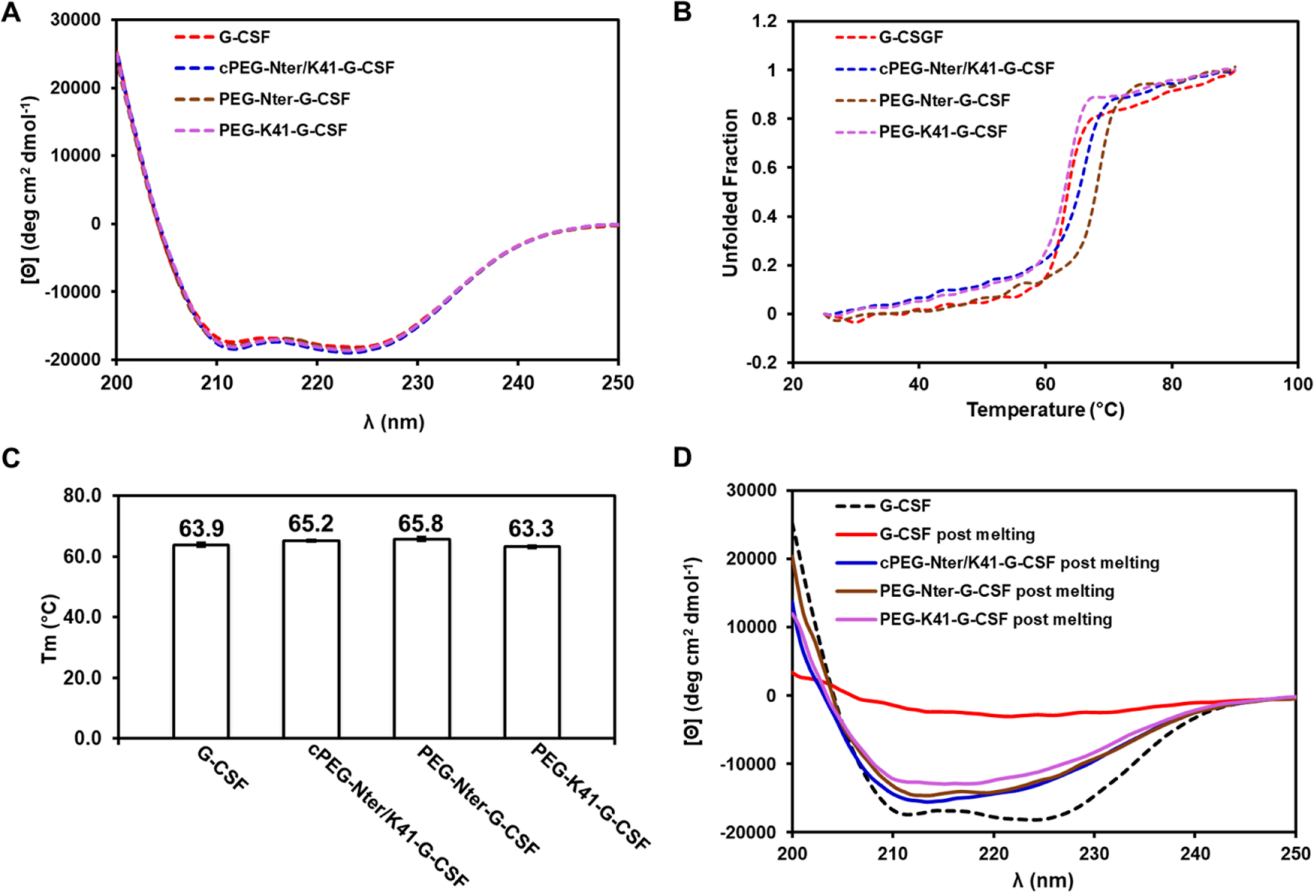
(A) CD spectra; (B) temperature dependence of the CD intensity at 222 nm of G-CSF; (C) melting temperatures of G-CSF, cPEG-Nter/K41-G-CSF, PEG-Nter-G-CSF, and PEG-K41-G-CSF; and (D) CD spectra at 25 °C after melting of G-CSF, cPEG-Nter/K41-G-CSF, PEG-Nter-G-CSF, and PEG-K41-G-CSF (continuous lines) compared to CD spectra of G-CSF in the native form (dashed line). The samples were dissolved in 10 mM acetate, 5% (v/v) sorbitol pH 4.6 buffer at a protein concentration of 0.1 mg/mL.

CD analysis was conducted to investigate if the site of attachment and the conformation of the polymer have an impact on the secondary structure of the G-CSF conjugates and its thermal stability. The structure of G-CSF is prevalently formed by four antiparallel helices connected by two long and one short hairpin-type loops^27^ that under spectroscopy analysis show the two characteristic negative bands at 208 and 222 nm.^28^ The effect of the polymer on the protein’s surface was evaluated in the far-UV, and as shown in Figure 4A, the dichroic profiles of the G-CSF and the conjugates were superimposable, indicating that no variation in the protein secondary structure occurred with all of the PEGylation strategies used.

The protein unfolding was evaluated by recording the ellipticity at 222 nm and increasing the temperature of the samples from 25 to 90 °C. The protein maintained its secondary structure up to a temperature ranging between 50 and 55 °C and rapidly lost its native structure at the melting temperatures (*T*_m_) of about 64 °C. The thermal stabilities of cPEG-Nter/K41-G-CSF and PEG-Nter-G-CSF were increased by 1.3 and 1.9 °C, respectively, compared to that of G-CSF. For PEG-K41-G-CSF, the stability was only slightly affected as the melting temperature decreased by ∼0.6 °C (Figure 4B). The Tm values, reported in Figure 4C, were calculated assuming that the protein was completely folded at 25 °C and completely unfolded at 90 °C.

The reversibility of the thermal unfolding was investigated by measuring the recovery of the CD signal of the G-CSF and the PEGylated conjugates after heating the solutions at 90 °C and cooling them back to 25 °C. The CD spectrum of G-CSF at 25 °C after melting showed the complete disappearance of the bands at 208 and 222 nm, meaning the loss of the native α-helical structure and a permanent denaturation. Interestingly, the same behavior was not recorded for the conjugates as the transition from the denatured form at 90 °C to back at 25 °C was found to be partially reversible for the conjugates, with slightly better results for the conjugates cPEG-Nter/K41-G-CSF and PEG-Nter-G-CSF (Figure 4D). As expected, for the circular conjugate cPEG-Nter/K41-G-CSF, the thermal stability and the reversibility of the thermal unfolding resulted in a combination of the effects seen for the linear counterparts.

### Pharmacokinetic Study in Rats

**Figure 5:**
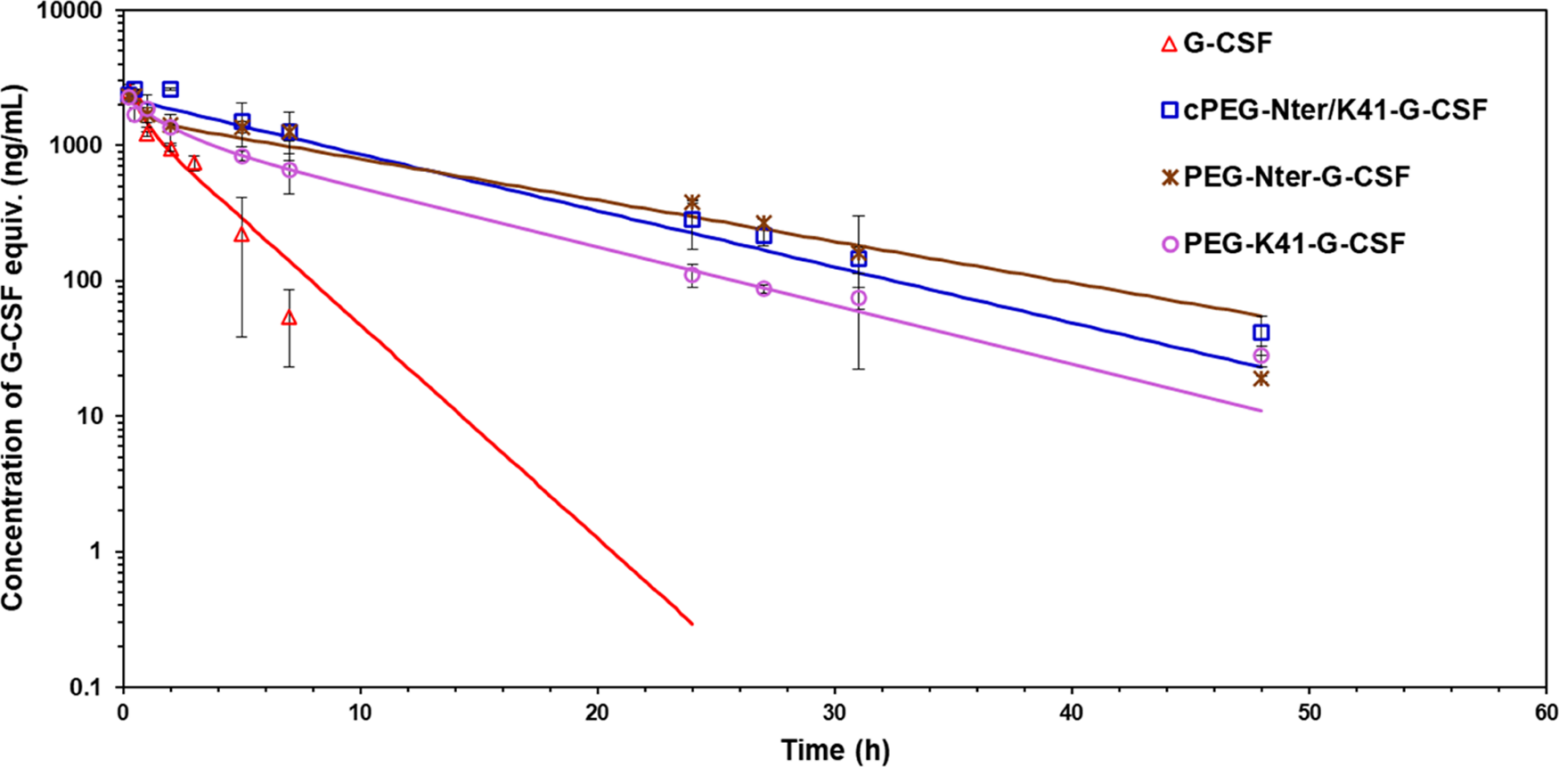
Pharmacokinetic profiles of G-CSF, cPEG-Nter/K41-G-CSF, PEG-Nter-G-CSF, and PEG-K41-G-CSF in Sprague–Dawley rats (3 per group) after i.v. administration of 100 μg/kg G-CSF (protein equivalent). Data are shown as average ± SD.

The pharmacokinetics of three G-CSF conjugates, cPEG-Nter/K41-G-CSF, PEG-Nter-G-CSF, and PEG-K41-G-CSF, were evaluated in rats after intravenous injection in the lateral tail vein. As depicted in Figure 5, the conjugates demonstrated a significant increase in the half-lives, with detectable levels of G-CSF up to 48 h, while the native protein fell below the detection limit after 7 h. The primary pharmacokinetic parameters are presented in Table 1. The half-lives of cPEG-Nter/K41-G-CSF, PEG-Nter-G-CSF, and PEG-K41-G-CSF were, respectively, 5.9-, 5.2-, and 3.7-fold higher than that of G-CSF. The circular conjugate, cPEG-Nter/K41-G-CSF, exhibited the longest half-life compared to the other conjugates.

**Table 1:** Main Pharmacokinetic Parameters of G-CSF, cPEG-Nter/K41-G-CSF, PEG-Nter-G-CSF, and PEG-K41-G-CSF after i.v. Administration of 100 μg/kg G-CSF (Protein Equivalent) in Sprague–Dawley Rats (*n* = 3)^a^

Compound	*t*_1/2α_ (h)	*t*_1/2β_ (h)	AUC 0-inf (ng·h/mL)	CL (mL/h)	Vβ (mL)
G-CSF	0.35 ± 0.04	1.9 ± 0.34	5743.5 ± 870.8	4.09 ± 0.47	11.27 ± 2.39
cPEG-Nter/K41-G-CSF	0.51 ± 0.71	11.19 ± 3.5**	24 123.6 ± 9562.5*	0.68 ± 0.27^#^	11.67 ± 7.8
PEG-Nter-G-CSF	0.37 ± 0.1	9.9 ± 1.28**	24 059.6 ± 5386.7*	1 ± 0.22^#^	14.1 ± 1.35
PEG-K41-G-CSF	0.52 ± 0.48	7.1 ± 1.13*	14 668.4 ± 2250.4	1.02 ± 0.17^#^	10.27 ± 0.63

a *t*_1/2α_ = half-life of distribution phase; *t*_1/2β_ = half-life of elimination phase; AUC = area under the curve; CL = clearance; Vβ = terminal volume. **p < 0.01 vs G-CSF, *p < 0.05 vs G-CSF, ^#^*p* < 0.0001 vs G-CSF.

In agreement with the prolonged half-lives, the clearance of the conjugates was significantly reduced in comparison to that of the native protein, especially for cPEG-Nter/K41-G-CSF. Additionally, PEGylation led to an enhancement of the bioavailability of the conjugates, with an increase in the area under the curve (AUC). The AUC for cPEG-Nter/K41-G-CSF and PEG-Nter-G-CSF was 4.2-fold higher than that of G-CSF, while PEG-K41-G-CSF showed a 2.6-fold increase in AUC, consistent with previous reports.^11^

Overall, the pharmacokinetic comparison of the conjugates demonstrated that the ring conformation in cPEG-Nter/K41-G-CSF resulted in a slightly more extended elimination half-life and a delayed clearance, although these values are not statistically significant with respect to those of the linear PEG-G-CSF conjugates.

### In Vivo Activity of PEG-G-CSF Conjugates

G-CSF specifically induces the proliferation and differentiation of neutrophil precursors upon binding to its specific cell-surface receptor, and it also regulates the survival of mature neutrophils. In this study, we evaluated the in vivo biological activity of cPEG-Nter/K41-G-CSF, PEG-Nter-G-CSF, and PEG-K41-G-CSF conjugates by comparing their effects on the levels of granulocytes and monocytes in mice after subcutaneous injections. Blood samples were collected at predetermined time points over 7 days after a single dose of the conjugates at 1 mg/kg of G-CSF equivalent. The native G-CSF was injected daily at the dose of 0.14 mg/kg and used as a positive control.

**Figure 6:**
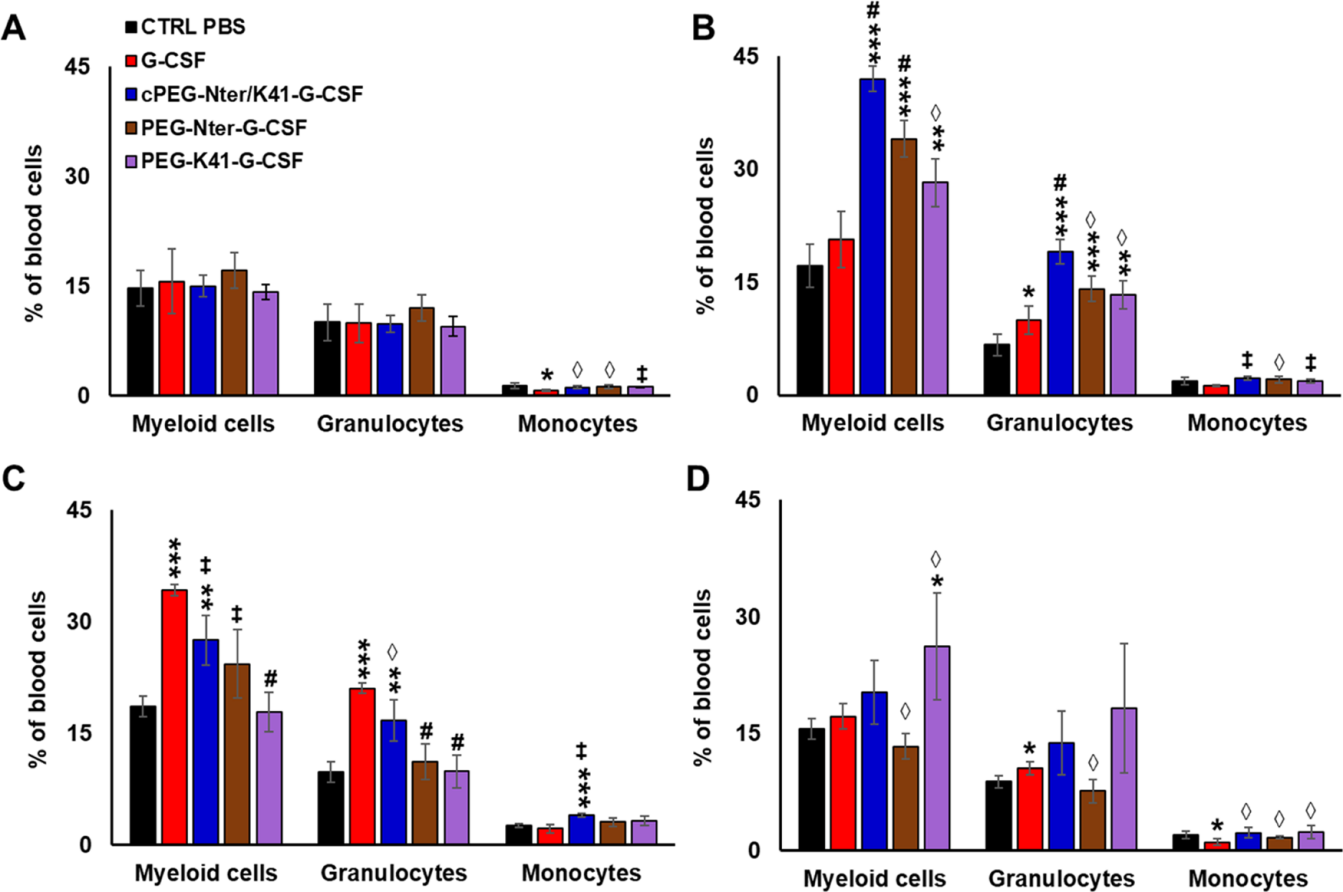
Levels of myeloid cells, granulocytes, and monocytes in the blood of C57BL/6 mice (*n* = 4 per group) at 1 day (A), 3 days (B), 5 days (C), and 7 days (D) post administration of a single dose (1 mg/kg, G-CSF equivalent) of cPEG-Nter/K41-G-CSF, PEG-Nter-G-CSF, or PEG-K41-G-CSF, or a daily administration (0.14 mg/kg, protein equivalent) of G-CSF. Vehicle (CTRL) was used as control. The data are presented as mean ± SD. The error bars reflect the SD. Symbols: *p < 0.05 vs control; **p < 0.01 vs control; ***p < 0.001 vs control; ^◊^*p* < 0.05 vs G-CSF; ^‡^*p* < 0.01 vs G-CSF; ^#^*p* < 0.001 vs G-CSF (significance was calculated using ANOVA). *p* > 0.05 if not indicated.

Our results showed that a single administration of all of the conjugates induced a significant increase in the levels of circulating myeloid cells, especially granulocytes, which was higher or comparable to that of the native cytokine injected daily (Figure 6). The quantification of myeloid cell sub-populations in the spleen also confirmed that a single injection of the conjugates showed comparable results with respect to daily injection of G-CSF (Figure S8). In the blood, the peak effect of neutrophil mobilization was observed on day 3 and gradually decreased in the following time points (Figure 6). Interestingly, the cPEG-Nter/K41-G-CSF conjugate achieved a bioactive response on days 3 and 5 that was superior to that of the linear PEGylated G-CSF derivatives in comparison to the G-CSF or to the control groups. This finding suggests that the circular conjugation did not interfere with the receptor-binding biological activity of the cytokine.

Moreover, the circular conjugate appeared to be faster in inducing the mobilization of myeloid cells. The higher potency of cPEG-Nter/K41-G-CSF is possibly due to the lower steric hindrance of the polymer after circular PEGylation. The PEG forms a closed ring with the protein, limiting its mobility and interfering less with the process of protein/receptor interaction with respect to the methods of linear conjugation. Therefore, this approach could be advantageous in the design of G-CSF conjugates for therapeutic applications.

## Conclusions

PEGylation is a well-established strategy for improving the therapeutic properties of biologics. The cPEG-Nter/K41-G-CSF is a new G-CSF derivative, which is a mono-PEGylated conjugate with both ends of the polymer chain linked to the protein in a ring-like conformation. This conformation results in a more compact hydrodynamic volume for the conjugate, as demonstrated by gel filtration analysis, which might enhance its ability to penetrate and diffuse through biological tissues and improve its therapeutic efficacy. Additionally, the 20 kDa PEG achieved an extension of blood circulation half-life that conferred a long-lasting effect that is superior or comparable to the daily administration of the reference cytokine.

Our results show that G-CSF circular PEGylation did not interfere with the biological activity of the cytokine and, in fact, showed a slightly better statistically significant activity with respect to other conjugates when that was compared to G-CSF or control groups. PEGylation approaches. This improvement may be due to the lower steric hindrance of the PEG chain linked to the protein in a ring mode. Overall, this approach provides a new tool for designing optimized polymer–protein conjugates with improved therapeutic efficacy.

## Supporting Information Available

Additional characterizations (PDF)
