# Supplementary material for: Improving the Therapeutic Potential of G-CSF through Compact Circular PEGylation Based on Orthogonal Conjugations
Source: Biomacromolecules. 2023 Aug 28;24(9):4229–39. doi: 10.1021/acs.biomac.3c00543 (PMC10498445; doi:10.1021/acs.biomac.3c00543)
Supplement: Supplementary file 1 — bm3c00543_si_001.pdf [file bm3c00543_si_001.pdf]

Improving the therapeutic potential of G-CSF through compact circular PEGylation based on orthogonal conjugations

*Antonella Grigoletto[1], Valentina Marotti[1], Tommaso Tedeschini[1], Benedetta Campara[1], Ilaria Marigo[2,3], Vincenzo Ingangi[3], Gianfranco Pasut[1]**

1. University of Padova, Dept. Pharmaceutical and Pharmacological Sciences, Via Marzolo 5, 35131, Padova, Italy.

2. Department of Surgery, Oncology and Gastroenterology, University of Padova, Padova, Italy

3. Istituto Oncologico Veneto IOV – IRCCS, Via Gattamelata 64, 35128, Padova, Italy

*Corresponding author:

gianfranco.pasut@unipd.it

Keywords: PEGylation, circular PEGylation, polymer conjugation, G-CSF, protein conjugate

SUPPORTING INFORMATION

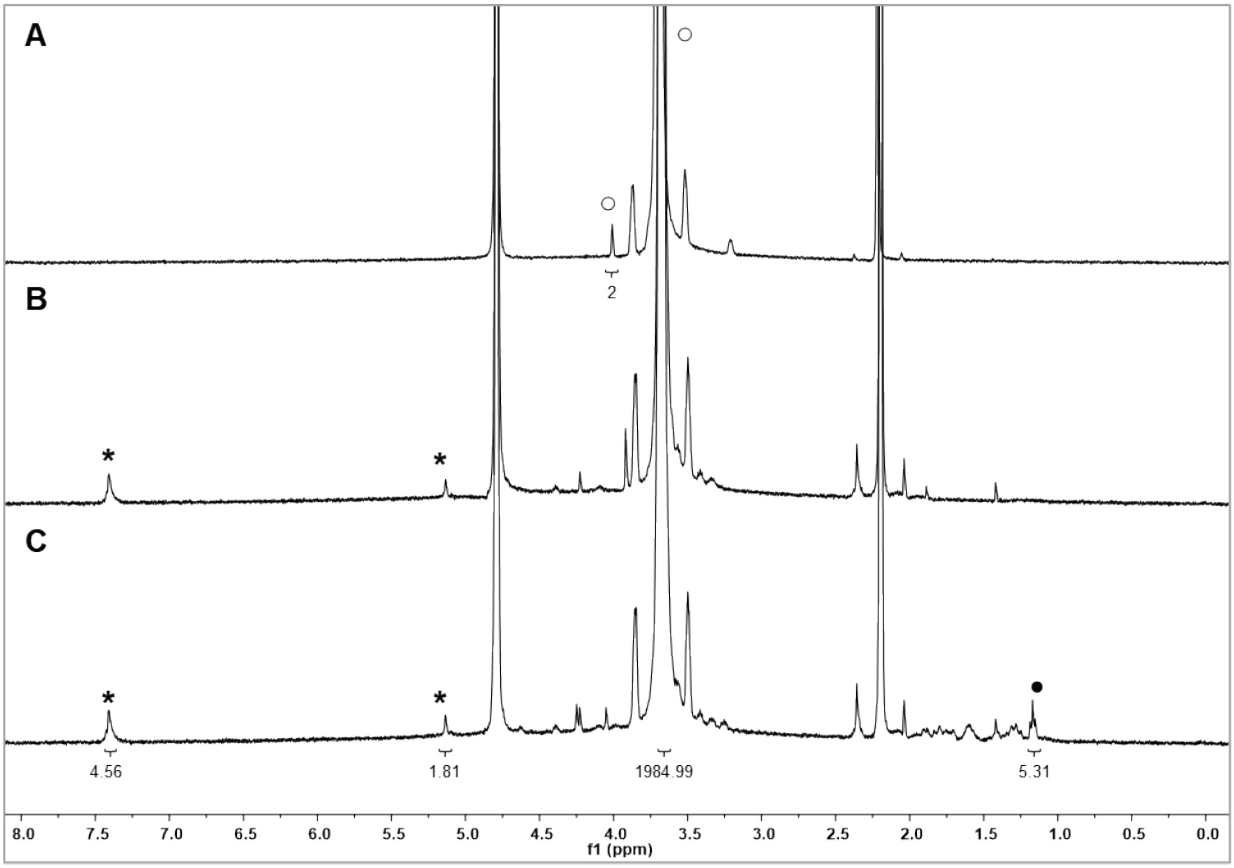

Figure S1. ^{1}H NMR of NH_2-PEG-COOH (A), ZQG-PEG_{20k}-COOH (B) and ZQG-PEG_{20k}-acetal (C) in D_2O; PEG (○): 4 (s, 2H), 3.7 (s, 1970H), ZQG (*): 7.41 (m, 5H), 5.13 (s, 2H), 4-aminobutyraldehyde diethyl acetal (•): 1.17 ppm (t, 6H).

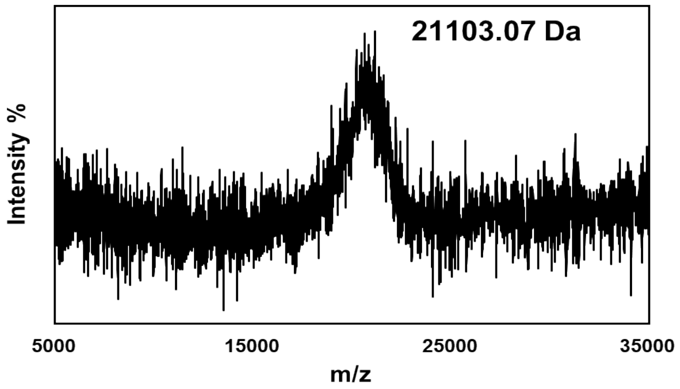

Figure S2. MALDI-TOF spectra of ZQG-PEG$_{20kDa}$-acetal.

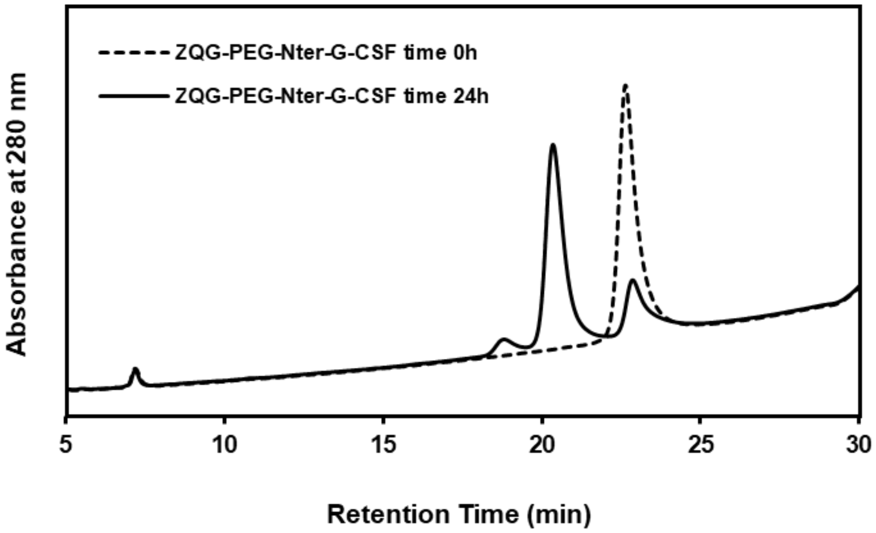

Figure S3. RP-HPLC analytical profile of G-CSF (dashed line) and reaction mixture of G-CSF with ZQG-PEG-aldehyde (continuous black line). G-CSF was eluted at 22.6 min, while at 20.3 min a new peak, corresponding to the conjugate, was formed over 24h.

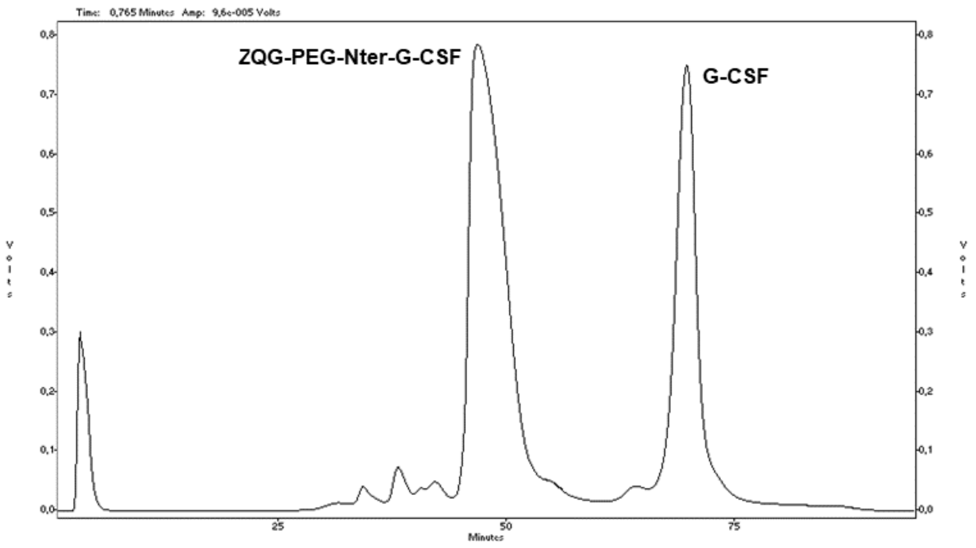

Figure S4. CEX-HPLC purification profile of ZQG-PEG-Nter-G-CSF. Elution was performed on a TSKgel SP-5PW column (7.5 × 75 mm; 10 µm) operating at a flow-rate of 1.0 ml/min with buffer A: 10 mM sodium phosphate pH 4.7 and buffer B: 100 mM sodium phosphate, 100 mM sodium chloride pH 4.85; gradient B%: 0’ 5%, 5’ 5%, 65’ 100%, 80’ 100%, 85’ 5%; the absorbance was recorded at 280 nm. The peak eluting at about 45 min corresponds to ZQG-PEG-Nter-G-CSF.

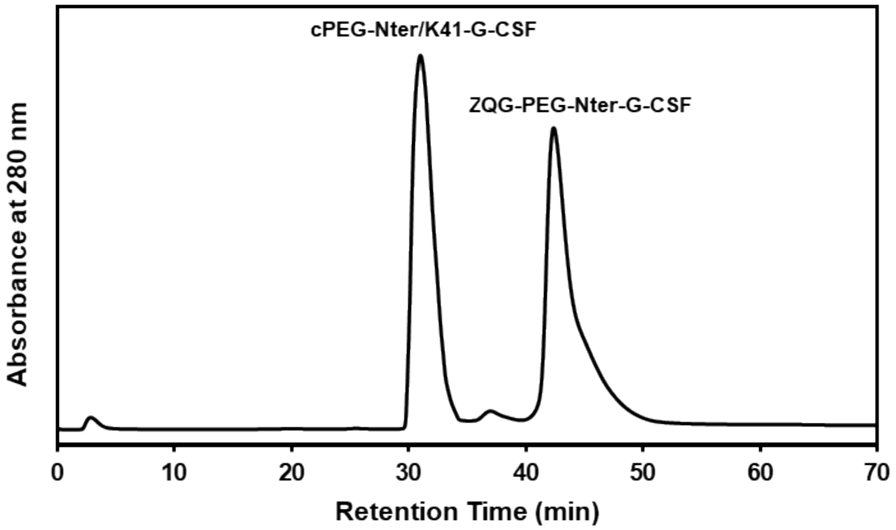

Figure S5. CEX-HPLC purification profile of cPEG-Nter/K41-G-CSF. Elution was performed on a TSKgel SP-5PW column (7.5 × 75 mm; 10 µm) operating at a flow-rate of 1.0 ml/min with buffer A: 10 mM sodium phosphate pH 4.7 and buffer B: 100 mM sodium phosphate, 100 mM sodium chloride pH 4.85; gradient B%: 0’ 5%, 5’ 5%, 65’ 100%, 80’ 100%, 85’ 5%; the absorbance was recorded at 280 nm. The peak eluting at about 30 min corresponds to cPEG-Nter/K41-G-CSF, while the peak eluting at about 45 min corresponds to ZQG-PEG-Nter-G-CSF.

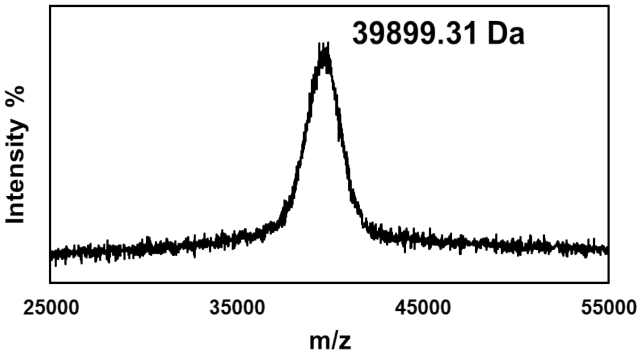

Figure S6. MALDI-TOF spectra of cPEG-Nter/K41-G-CSF.

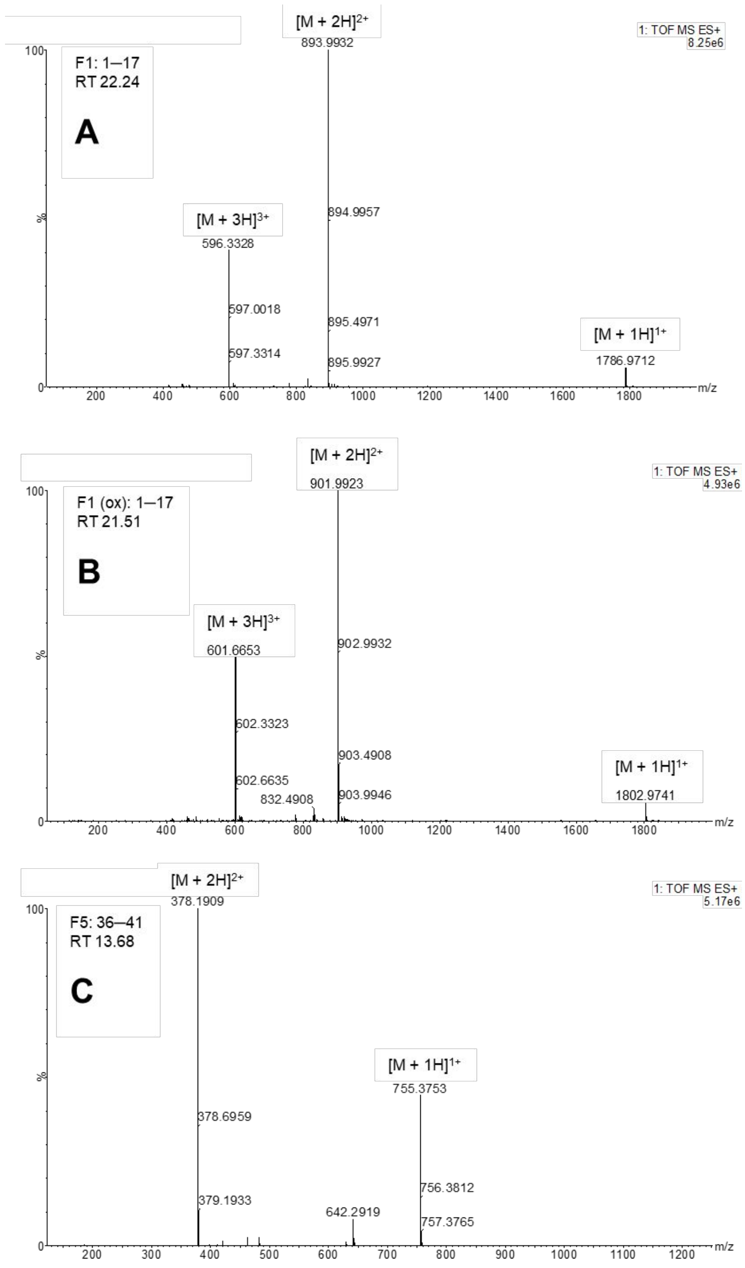

Figure S7. MS spectra of peptide F1 and F1 (ox) 1−17 (A, B) and peptide F5 36−41 (C).

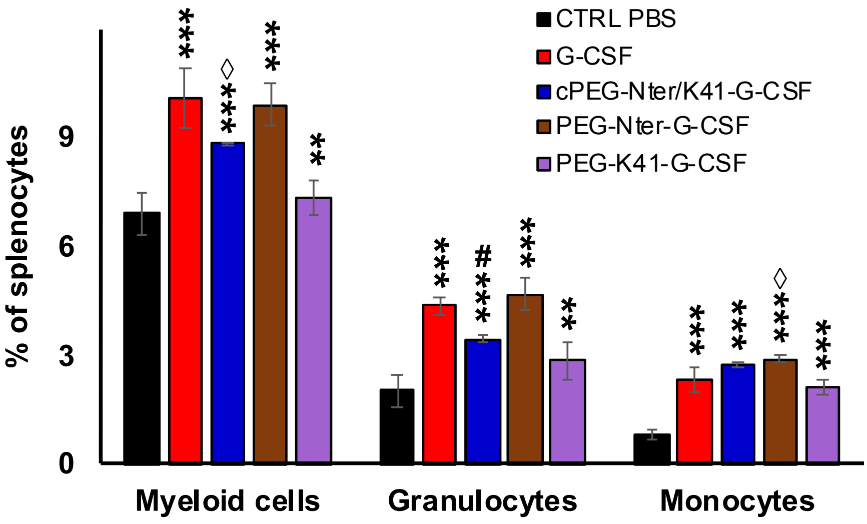

	p < 0.001	p < 0.01	p < 0.05
**vs. CTRL**	***	**	*
**vs. G-CSF**	#	‡	◊

Figure S8. Quantification of myeloid cells sub-populations in the spleen.

Table S1. Tryptic peptides of native G-CSF obtained upon digestion with trypsin. Molecular masses were determined by LC-MS^E.

Sequence	Fragment	Start	End	RT (Min)	Calculated Peptide Mass	Measured Peptide Mass	b/y possible	b/y found	Intensity (Counts)
MTPLGPASSLPQSFLLK	F1	1	17	22.24	1785.9698	1785.9696	32	31	6531965
**M**TPLGPASSLPQSFLLK	F1 (ox)	1	17	21.51	1801.9647	1801.9662	32	30	4118851
CLEQVR	F2	18	23	13.85	803.3960	803.3967	10	5	380167
CLEQVRK	F2-3	18	24	12.78	931.4910	931.4946	12	6	45154
KIQGDGAALQEK	F3-4	24	35	13.51	1256.6725	1256.6744	22	20	1095538
IQGDGAALQEK	F4	25	35	14.0	1128.5775	1128.5796	20	18	2311984
IQGDGAALQEKLCATYK	F4-5	25	41	20.15	1864.9353	1864.9686	32	2	12504
LCATYK	F5	36	41	13.68	754.3684	754.3695	10	9	1509958
RAGGVLVASHLQSFLEVSYR	F7-8	148	167	20.35	2188.1753	2188.1746	38	4	4810
AGGVLVASHLQSFLEVSYR	F8	149	167	21.51	2032.0741	2032.0724	36	5	4548
VLR	F9	168	170	10.69	386.2641	386.2634	4	3	99917
HLAQP	F10	171	175	12.98	564.3020	564.3027	8	8	457060

Table S2. Tryptic peptides of ZQG-PEG-Nter-G-CSF obtained upon digestion with trypsin. Molecular masses were determined by LC-MS^E.

Sequence	Fragment	Start	End	RT (Min)	Calculated Peptide Mass	Measured Peptide Mass	b/y possible	b/y found	Intensity (Counts)
MTPLGPASSLPQSFLLK	F1	1	17	22.16	1785.9698	1785.9675	32	21	109671
**M**TPLGPASSLPQSFLLK	F1 (ox)	1	17	21.47	1801.9647	1801.9631	32	18	77024
CLEQVR	F2	18	23	13.71	803.3960	803.3980	10	8	1035261
CLEQVRK	F2-3	18	24	12.64	931.4910	931.4965	12	8	114774
KIQGDGAALQEK	F3-4	24	35	13.37	1256.6725	1256.6750	22	21	2103453
IQGDGAALQEK	F4	25	35	13.92	1128.5775	1128.5809	20	19	2821615
IQGDGAALQEKLCATYK	F4-5	25	41	20.12	1864.9353	1864.9714	32	1	2715
LCATYK	F5	36	41	13.58	754.3684	754.3709	10	10	2974252
RAGGVLVASHLQSFLEVSYR	F7-8	148	167	20.28	2188.1753	2188.1790	38	22	75762
AGGVLVASHLQSFLEVSYR	F8	149	167	21.47	2032.0741	2032.0776	36	21	90045
VLR	F9	168	170	10.52	386.2641	386.2641	4	3	171741
HLAQP	F10	171	175	12.85	564.3020	564.3038	8	8	814857

Table S3. Tryptic peptides of cPEG-Nter/K41-G-CSF obtained upon digestion with trypsin. Molecular masses were determined by LC-MS^{E}.

Sequence	Fragment	Start	End	RT (Min)	Calculated Peptide Mass		Measured Peptide Mass	b/y possible	b/y found	Intensity (Counts)
MTPLGPASSLPQSFLLK	F1	1	17	22.15	1785.9698		1785.9651	32	8	29169
**M**TPLGPASSLPQSFLLK	F1 (ox)	1	17	21.44	1801.9647		1801.9572	32	13	22748
CLEQVR	F2	18	23	13.68	803.3960		803.3969	10	10	557457
CLEQVRK	F2-3	18	24	12.58	931.4910		931.4934	12	6	57402
KIQGDGAALQEK	F3-4	24	35	13.37	1256.6725		1256.6749	22	22	737943
IQGDGAALQEK	F4	25	35	13.88	1128.5775		1128.5797	20	17	1102380
IQGDGAALQEKLCATYK	F4-5	25	41	20.1	1864.9353		-	-	-	-
LCATYK	F5	36	41	13.54	754.3684		754.3686	10	0	3432
RAGGVLVASHLQS FLEVSYR	F7-8	148	167	20.25	2188.1753		2188.1768	38	25	161571
AGGVLVASHLQS FLEVSYR	F8	149	167	21.44	2032.0741		2032.0767	36	27	173737
VLR	F9	168	170	10.49	386.2641		386.2639	4	3	104423
HLAQP	F10	171	175	12.81	564.3020		564.3028	8	7	429330

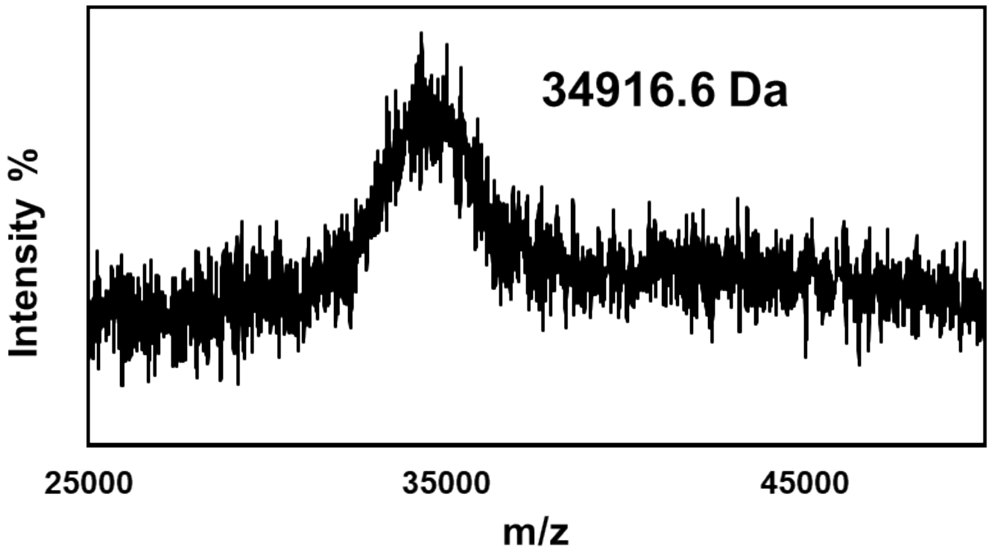

Figure S8. MALDI-TOF spectrum of the product (F1)-PEG-(F5-6) obtained by digestion of cPEG-Nter/K41-G-CSF with trypsin.
